# Exploring the pathogenesis of chronic atrophic gastritis with atherosclerosis via microarray data analysis

**DOI:** 10.1097/MD.0000000000037798

**Published:** 2024-04-19

**Authors:** Xiaoxiao Men, Xiuju Shi, Qianqian Xu, Mingyue Liu, Hongli Yang, Ling Wang, Xiaoju Men, Hongwei Xu

**Affiliations:** a Department of Gastroenterology, Shandong Provincial Hospital, Cheeloo College of Medicine, Shandong University, Jinan, China; b Department of Gastroenterology, Shandong Provincial Hospital Affiliated to Shandong First Medical University, Jinan, China; c Hunan Key Laboratory of the Research and Development of Novel Pharmaceutical Preparations, Changsha Medical University, Changsha, PR China.

**Keywords:** Atherosclerosis, atrophic gastritis, hub genes, transcription factors

## Abstract

Although several studies have reported a link between chronic atrophic gastritis (CAG) and atherosclerosis, the underlying mechanisms have not been elucidated. The present study aimed to investigate the molecular mechanisms common to both diseases from a bioinformatics perspective. Gene expression profiles were obtained from the Gene Expression Omnibus database. Data on atherosclerosis and CAG were downloaded from the GSE28829 and GSE60662 datasets, respectively. We identified the differentially expressed genes co-expressed in CAG and atherosclerosis before subsequent analyses. We constructed and identified the hub genes and performed functional annotation. Finally, the transcription factor (TF)-target genes regulatory network was constructed. In addition, we validated core genes and certain TFs. We identified 116 common differentially expressed genes after analyzing the 2 datasets (GSE60662 and GSE28829). Functional analysis highlighted the significant contribution of immune responses and the positive regulation of tumor necrosis factor production and T cells. In addition, phagosomes, leukocyte transendothelial migration, and cell adhesion molecules strongly correlated with both diseases. Furthermore, 16 essential hub genes were selected with cytoHubba, including *PTPRC, TYROBP, ITGB2, LCP2, ITGAM, FCGR3A, CSF1R, IRF8, C1QB, TLR2, IL10RA, ITGAX, CYBB, LAPTM5, CD53, CCL4*, and *LY86.* Finally, we searched for key gene-related TFs, especially SPI1. Our findings reveal a shared pathogenesis between CAG and atherosclerosis. Such joint pathways and hub genes provide new insights for further studies.

## 1. Introduction

Chronic inflammation is a hallmark of several conditions, such as rheumatoid arthritis, chronic atrophic gastritis (CAG), inflammatory bowel disease, atherosclerosis, and cancer.^[1–3]^ Among these conditions, CAG is highly prevalent as a chronic inflammation of the gastric mucosa, including gastric mucosal atrophy and glandular cytopenia.^[4]^ Similarly, atherosclerosis is associated with chronic inflammation of the arteries with lipid accumulation and plaque formation.^[5,6]^ Studies have reported that atrophic gastritis is associated with atherosclerosis regardless of *Helicobacter pylori* status.^[7–10]^

Atrophic gastritis can cause hyperhomocysteinemia, an independent risk factor for atherosclerosis.^[8]^ The 2 conditions include overlapping inflammatory environmental factors, including IL1B,^[11,12]^ IL1RN,^[13,14]^ PTGS2,^[15,16]^ IL1A,^[17,18]^ MUC1,^[19–21]^ IL10,^[22–24]^ S100A8,^[25,26]^ and GSTM1.^[27,28]^ In addition, multiple signaling pathways are involved in both diseases. For example, Nrf2-ARE signaling is involved in alleviating inflammation-related pathogenesis, such as gastritis and atherosclerosis.^[29]^ Free radical stress leads to tissue damage and disease progression in atherosclerosis and gastritis.^[30,31]^ However, these results were mainly from serological or pathological studies and failed to reveal genomic relationship between CAG and atherosclerosis.

Bioinformatics analysis can provide new insights into the pathogenesis of diseases by identifying potential pivotal molecules and information pathways. The present study explored CAG complicated by atherosclerosis using a bioinformatics approach to provide new clues about the combined mechanisms of the 2 diseases at the genetic level.

## 2. Materials and methods

### 2.1. Data source

Microarray materials were collected from the Gene Expression Omnibus database of the NCBI (http://www.ncbi.nlm.nih.gov/geo/). GSE60662 and GSE28829 were used as the training datasets, and GSE60427 and GSE100927 were used as the validation datasets. In detail, we selected 7 CAG and 4 adjacent normal tissue samples from the GSE60662 dataset. For atherosclerosis, 12 control samples and 14 samples of advanced atherosclerotic (AA) plaques were selected from the GSE28829 dataset.

### 2.2. Identification of differentially expressed genes (DEGs)

Differential gene expression analysis was performed with the R package limma (version 3.44.1). We obtained the expression profile dataset, performed multiple linear regression analysis using the lmFit function, calculated the statistics using the eBays function, calculated the logarithm of differential expression by empirical Bayes methods, and eventually obtained the significance of differences for each gene. FDR < 0.05 and fold change > 2 were used as thresholds. The results of DEG in the GSE60662 and GSE28829 datasets are shown in the heat map. Finally, the DEGs from both datasets were intersected with a Venn diagram using the online tool Jvenn (http://jvenn.toulouse.inra.fr/app/index.html).

### 2.3. Enrichment analyses of the DEGs

Functional enrichment analysis of DEGs with the same expression trends in both datasets was done using the Database for Annotation, Visualization, and Integrated Discovery (DAVID). DAVID is an online database that helps to integrate and visualize biological functions and protein lists^[32]^ and can be used for gene ontology (GO) and Kyoto Encyclopedia of Genes and Genomes (KEGG) analysis. GO is a database established by the Gene Ontology Consortium to comprehensively analyze the attribution of genes and gene products in organisms, including molecular functions (MF), biological processes (BP), and cellular components (CC). KEGG is a database resource for understanding the high-level functions and utility of biological systems from genomic and molecular-level information. It comprises molecular building blocks of genomic and chemical information that are combined with molecular wiring diagrams of interactions, reactions, and relational networks. FDR < 0.05 for enrichment analysis was considered significant, and the results have been demonstrated using the GOplot package.

### 2.4. Protein–protein interaction (PPI) module analysis

The core functional modules of the differential genes were analyzed using the molecular complex assay (MCODE) plugin in Cytoscape.^[33]^ The selection criteria were set to k-core = 2, degree cutoff = 2, max depth = 100, and node score cutoff = 0.2. The obtained functional module-related genes were subjected to functional enrichment analysis using Metascape (http://metascape.org/gp/index.html#/main/step1). Metascape is a web tool that enables gene enrichment analysis, protein interaction network analysis, and many other actions. The site integrates more than 40 gene function annotation databases and provides diverse visualizations through which gene function can be easily explored and analyzed. The results of our study are presented through the enrichment network. We performed GO and KEGG analysis using Metascape.

### 2.5. Selection and analysis of hub genes

Hub genes were selected using cytoHubba in Cytoscape. The top 20 genes were obtained based on the built-in algorithms of MCC, DMNC, Degree, and EPC. The intersection of these 4 groups of genes was considered the hub genes. Subsequently, the co-expression networks of hub genes were constructed based on GeneMANIA, a Cytoscape plugin, to analyze gene list functions and identify internal associations.^[34]^ GeneMANIA results for hub genes were visualized using Cytoscape and chord diagram.

### 2.6. Validation of hub gene expression in other datasets

The GSE60427 and GSE100927 datasets were used to verify the expression of the identified hub genes. Ten CAG and 8 normal samples were obtained from the GSE60427 dataset. Additionally, 69 AA plaques and 35 control samples were obtained from the GSE100927 dataset. The comparison between the health and disease groups was performed using the *t* test, and the results are presented on a violin plot. *P* value < .05 was considered significant.

### 2.7. Prediction and verification of TFs

ChEA3 transcription factor (TF) enrichment analysis provides a platform (http://maayanlab.cloud/chea3/) to predict common TFs in multiple genes and identify potential TFs that may correlate with the list of regulatory genes. TFs are prioritized based on the overlap between the user-entered gene set and the TF target annotation set stored within the ChEA3 database. Finally, we validated the expression level of the core TFs in the dataset using a *t* test. *P* value < .05 was considered significant.

### 2.8. Ethics statement

This study was conducted using public database data, so ethical and consent permission is unnecessary.

## 3. Results

### 3.1. Identification of DEGs

**Figure 1.**
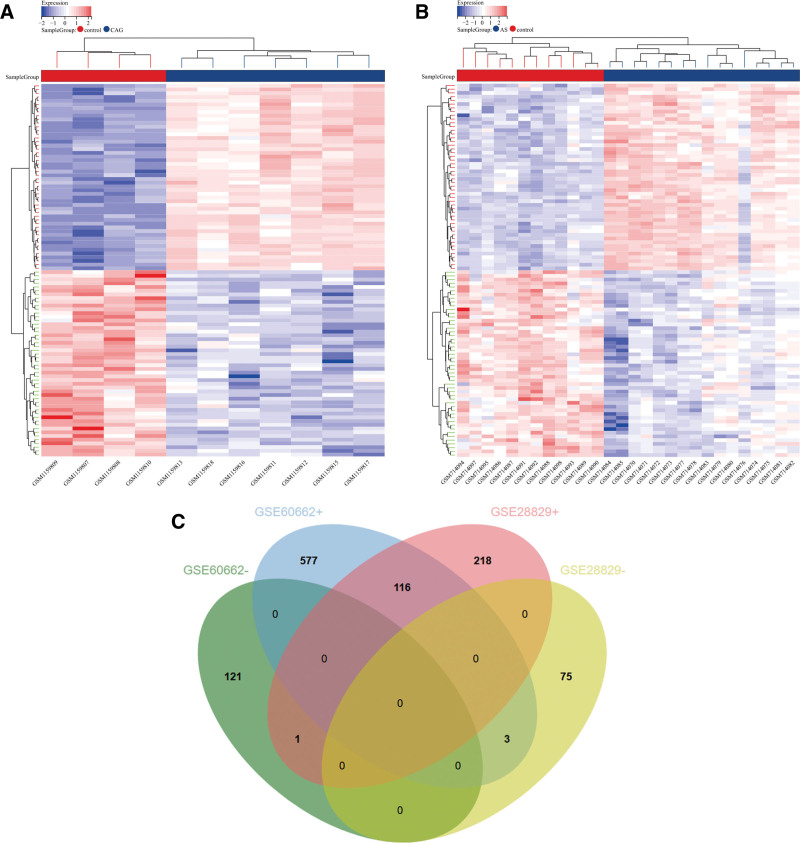
Heat map diagram and Venn diagram. (A) The heat map of GSE60662. (B) The heat map of GSE28829. Upregulated genes are marked in red; downregulated genes are marked in blue. (C) The 2 datasets showed downregulated DEGs (−) and upregulated DEGs (+).

After standardizing the microarray results, we identified a total of 819 DEGs in the GSE60662 datasets (Fig. 1A) and 413 DEGs in the GSE28829 datasets (Fig. 1B), respectively. Each dataset’s heat maps of DEGs were created using the R software package Pheatmap V1.0.12. A Venn diagram of both datasets showed 120 overlapping DEGs (Fig. 1C). Finally, we identified 116 DEGs after excluding genes with opposite expression trends.

### 3.2. Analysis of DEGs

**Figure 2.**
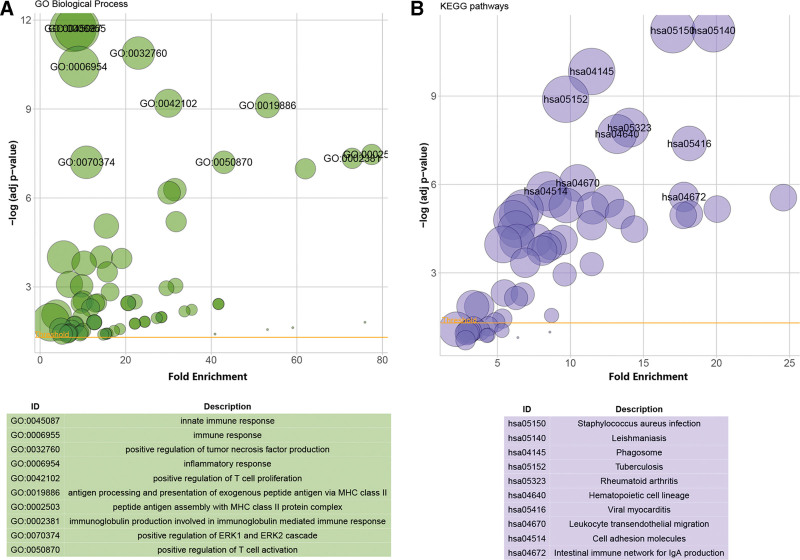
Enrichment analysis of DEGs. (A) The enrichment analysis results of GO. (B) The enrichment analysis results of KEGG pathway. The size of the circle represents the number of genes involved. Adjusted *P* value < .05 was considered significant.

Enrichment analysis of the 116 DEGs was performed using the DAVID online analysis tool. In terms of BP, the DEGs were responsible for 71 terms and were significantly enriched in immune responses (FDR = 1.93E-12), positive regulation of tumor necrosis factor production (FDR = 1.30E-11), positive regulation of T-cell proliferation (FDR = 6.38E-10) and activation (FDR = 6.27E-08), inflammatory responses (FDR = 3.74E-11), and positive regulation of the ERK1 and ERK2 cascade (FDR = 6.27E-08) (Fig. 2A). The analysis of the KEGG pathway revealed that the DEGs were highly enriched in 43 terms, such as phagosomes (FDR = 1.47E-10), leukocyte transendothelial migration (FDR = 8.92E-07), and cell adhesion molecules (FDR = 1.70E-06) (Fig. 2B). In addition, fluid shear stress and atherosclerosis, lipopolysaccharide-mediated signaling pathways, and several interleukins were involved in the BP. Similarly, immune cells, such as phagocytes, neutrophils, B cells, and T cells, were involved in the BP.

### 3.3. Construction and enrichment analysis of the PPI Module

**Figure 3.**
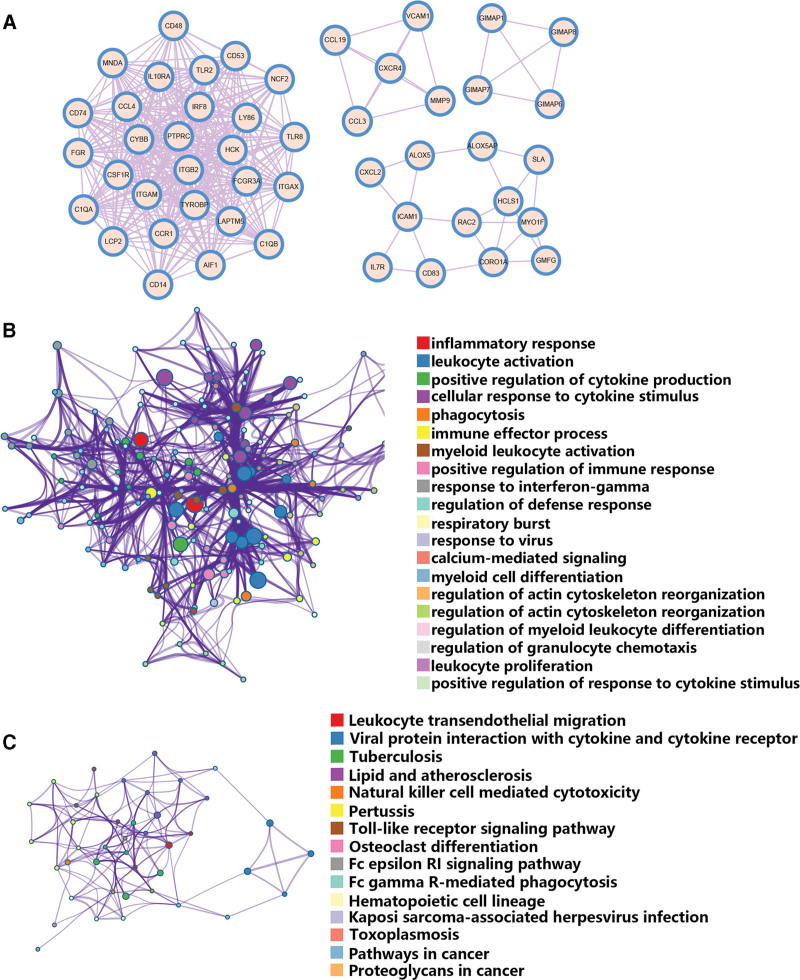
Significant gene module and enrichment analysis of the modular genes. (A) Four significant gene clustering modules. (B) GO enrichment analysis of the modular genes. (C) KEGG enrichment analysis of the modular genes. The size of the circle represents the number of genes involved.

Four tightly linked gene modules, comprising 49 common DEGs and 377 interaction pairs, were obtained using the MCODE plugin in Cytoscape (Fig. 3A). Functional enrichment analysis of the 49 common DEGs was performed using the Metascape online tool. GO analysis revealed that 49 common DEGs were associated with leukocyte activation, inflammatory responses, phagocytosis, cellular responses to cytokine stimulation and positive regulation of cytokine production (Fig. 3B). The KEGG pathway analysis revealed that 49 common DEGs were mainly involved in leukocyte transendothelial migration, the NF-kappa B signaling pathway, lipid and atherosclerosis, the Toll-like receptor signaling pathway, TNF signaling pathway, and viral protein interactions with cytokines and cytokine receptors (Fig. 3C). In addition, VCAM1, NCF2, RAC2, MMP9, and ICAM1 were associated with fluid shear stress and atherosclerosis.

### 3.4. Selection and analysis of hub genes

**Figure 4.**
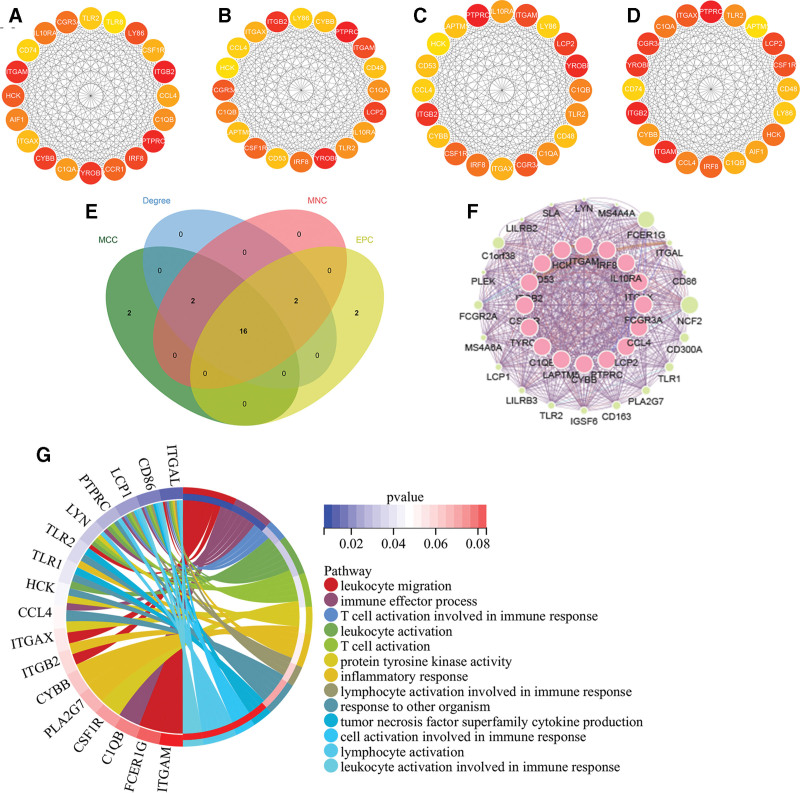
Venn diagram and co-expression network of hub genes. (A–D) Top 20 genes for the 4 algorithms obtained by cytohubba (MCC, Degree, MNC, EPC). (E) The Venn diagram showed that 4 algorithms have screened out 16 overlapping hub genes. (F) Hub genes and their co-expression network via GeneMANIA. (G) Enrichment analysis via GeneMANIA.

We identified the top 20 DEGs based on the 4 algorithms of cytoHubba (MCC, MNC, DMNC, Degree, and EPC) (Fig. 4A–D). Based on the intersection of the Venn diagrams of the 4 groups of genes, we detected 16 common hub genes, including *PTPRC, TYROBP, ITGB2, LCP2, ITGAM, FCGR3A, CSF1R, IRF8, C1QB, TLR2, IL10RA, ITGAX, CYBB, LAPTM5, CD53, CCL4*, and *LY86* (Fig. 4E). We identified the expression network and associated features of hub genes based on the Cytoscape plugin GeneMANIA. Sixteen hub genes showed a comprehensive PPI network with 0.33% physical interactions, 10.96% co-localization, 4.55% prediction, 83.37% co-expression, and 0.78% pathways (Fig. 4F). Gene enrichment analysis was performed on the obtained results. The hub genes were extensively associated with leukocyte migration, immune effector processes, and leukocyte and T-cell activation (Fig. 4G).

### 3.5. Validation of the expression of hub genes

**Figure 5.**
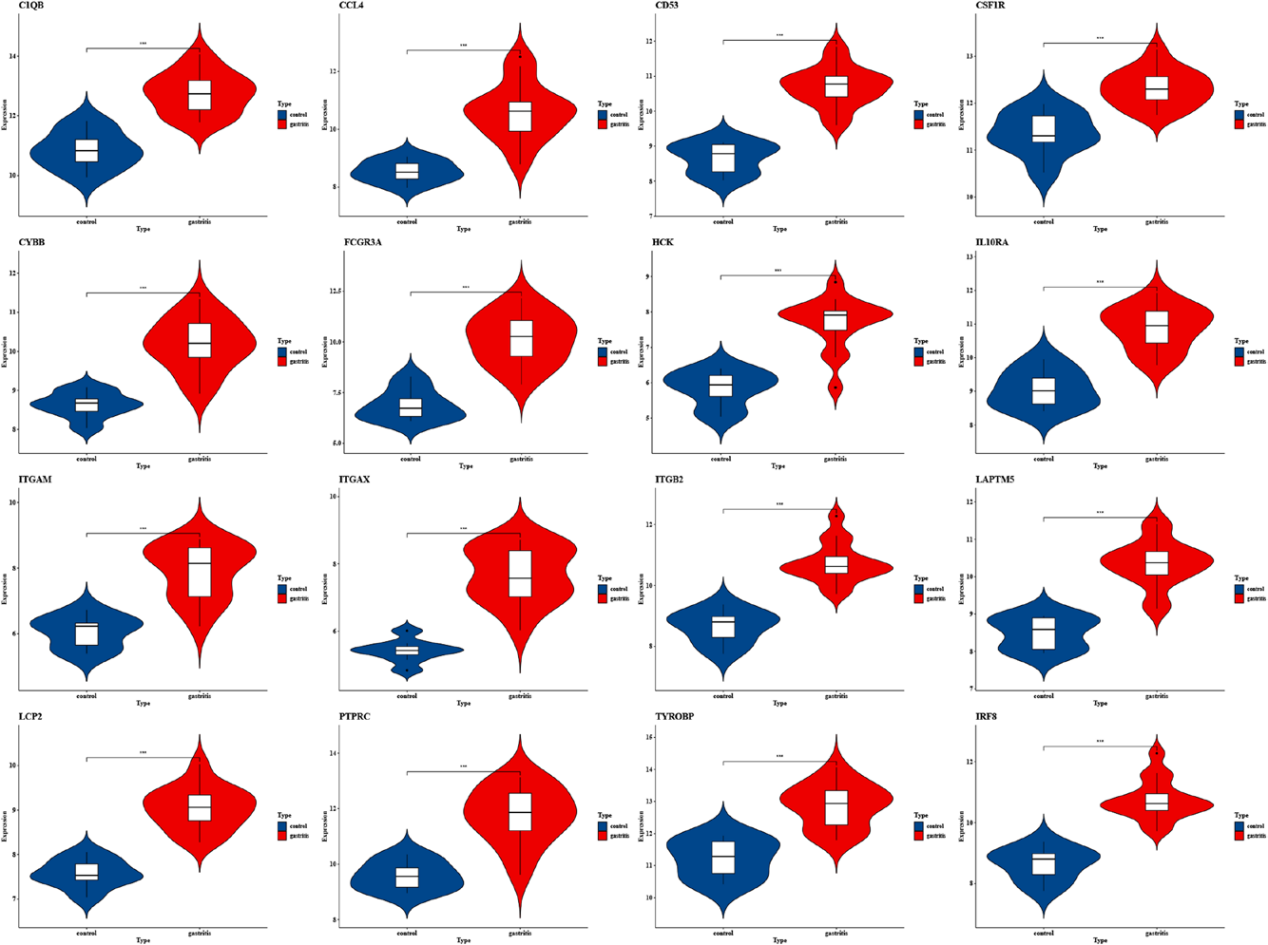
The expression level of hub gene in GSE60662. The comparison between the 2 sets of data uses the mean *t* test. *P* value < .05 was considered statistically significant. *P < .05; ***P < .001; ****P < .0001.

**Figure 6.**
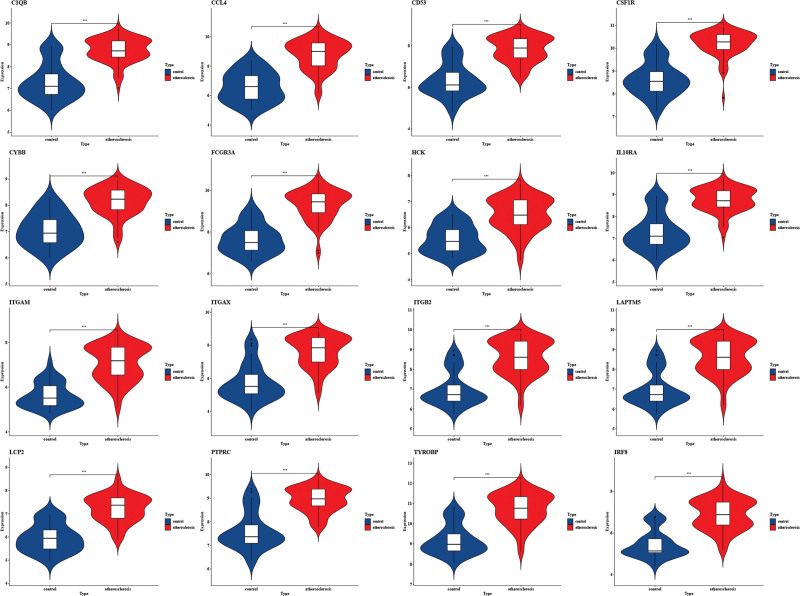
The expression level of hub gene in GSE28829. The comparison between the 2 sets of data uses the mean *t* test. *P* value < .05 was considered statistically significant. *P < .05; ***P < .001; ****P < .0001.

We confirmed the accuracy of the expression levels of hub genes using 2 additional validation datasets (GSE60427 and GSE100927). Violin plot results revealed that 16 hub genes were significantly upregulated in the CAG group (Fig. 5). Similarly, the expression of hub genes was higher in atherosclerotic plaques, as seen in the GSE100927 dataset (Fig. 6).

### 3.6. Prediction and verification of TFs

**Figure 7.**
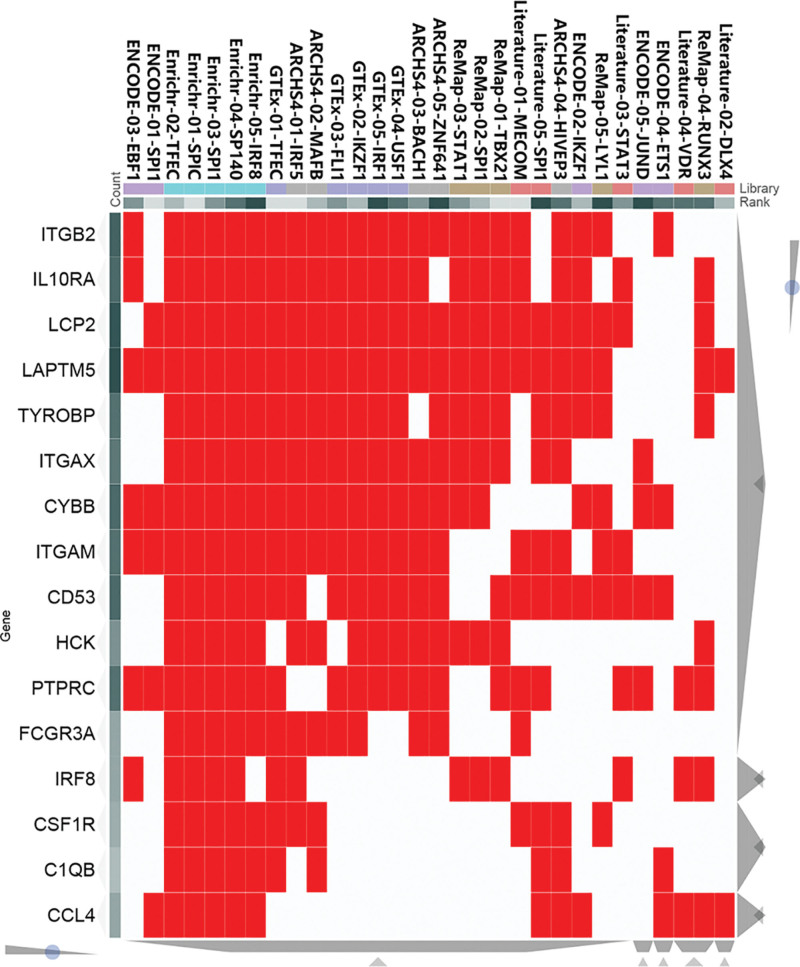
The clustergram between hub genes and TF from ChEA3 database.

**Figure 8.**
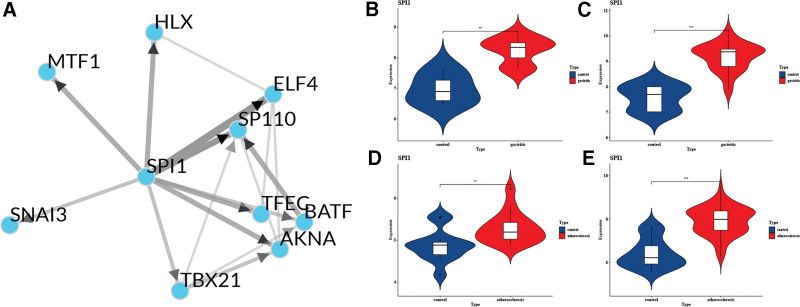
TF co-regulatory networks and the expression level of SPI1. (A) TF co-regulatory networks. (B–E) The expression level of SPI1 in GSE60427, GSE60662, GSE100927, and GSE28829. The comparison between the 2 sets of data uses the mean *t* test. *P* value < .05 was considered statistically significant. *P < .05; ***P < .001; ****P < .0001.

Based on the ChEA3 database, we identified the TFs associated with the 16 hub genes. The regulatory relationship between the top 10 TFs (*TFEC, SPI1, SP110, AKNA, MTF1, ELF4, TBX21, HLX, BATF*, and *SNAI3*) and hub genes is shown in Figure 7. In Figure 8A, the TF co-regulatory networks reveal that SPI1 is at the core of the TF regulatory system and is critical to the overall regulatory network. In addition, SPI1 is associated with all 16 central genes, such as HCK, ITGB2, ITGAM, and ITGAX. Further validation revealed that the core TF SPI1 was highly expressed in all 4 of the selected datasets of disease samples (Fig. 8B–E).

## 4. Discussion

The association between atherosclerosis and various diseases has been extensively demonstrated in numerous previous studies. Furthermore, through bioinformatics analysis, novel candidate genes have been identified as potential biomarkers or therapeutic targets, offering a new avenue for investigating the correlation between CAG and atherosclerosis. The common pathophysiological mechanisms between CAG and atherosclerosis continue to attract strong research interest. Previous studies have reported that atrophic gastritis might contribute to atherosclerosis in different ways, such as pulse wave velocity,^[35]^ ghrelin,^[36]^ and homocysteine.^[37]^ CAG can cause an increase in pulse wave velocity, which is an early preclinical marker of atherosclerosis.^[38]^ Ghrelin is a peptide hormone that has a protective effect against atherosclerosis.^[39]^ It inhibits proinflammatory responses and nuclear factor-kappaB activation in human endothelial cells.^[40]^ However, atrophic gastritis can lead to a decrease in its synthesis and secretion.^[41]^ In addition, there is a positive correlation between elevated serum homocysteine levels and gastric atrophy scores.^[42]^ Elevated homocysteine levels can alter endothelial integrity and tone via endothelial injury, VSMC proliferation, increasing the levels of ROS and inducing calcification by bone-related markers, such as OPN and OPG, ultimately leading to atherosclerosis.^[43–46]^ These findings have deepened our understanding of the correlation between the 2 conditions. To explore further at the genetic level, we investigated CAG and atherosclerosis comorbidity hypothesis by integrating data from public databases to identify common mechanisms of atrophic gastritis and atherosclerosis at genetic level for the first time.

Based on our analysis, we identified the links between CAG and atherosclerosis. The shared common DEGs and intrinsic mechanisms. In the present study, we detected 116 overlapping DEGs (all upregulated), including 49 modular genes and 16 hub genes. Functional enrichment analysis revealed significant enrichment in leukocytes, such as phagocytes, neutrophils, B cells, and T cells in CAG and atherosclerosis. This was consistent with previous studies. The previous bioinformatics analyses have demonstrated the significant involvement of MI macrophages in various diseases associated with atherosclerosis.^[47]^ Our results have shown there was a large influx of immune cells into the gastric mucosa, including macrophages and lymphocytes in CAG caused by *H. pylori*. M1 macrophages can release high levels of proinflammatory cytokines and inhibit acid secretion, leading to atrophic gastritis and parietal cell atrophy.^[48,49]^ They also play an important role in atherosclerosis. Proinflammatory leukocytes preferentially adhere to the activated endothelial monolayer overlying the early atherosclerotic plaques.^[50]^ Mononuclear phagocytes residing in the intima eventually accumulate cholesteryl esters and form foam cells, the hallmark of atherosclerotic lesions.^[50]^ CD4 T cells cause gastritis through a Th1-mediated immune response and can induce autoreactive inflammation against parietal cells, leading to atrophic gastritis and metaplasia.^[51,52]^ T cells promote atherosclerosis by producing cytokines such as IFN-γ, IL-2, and IL-17.^[53]^ Mast cells, B lymphocytes, and their associated cytokines, such as IL-6 and IFN-γ may aggravate the development of atherosclerosis.^[53]^ These studies support the results of our bioinformatics analysis and suggest a nonnegligible role of migration and differentiation of immune cells in these 2 conditions.

In addition, GeneMANIA analysis of 16 hub genes suggested an important role for protein tyrosine kinase activity. In *H. pylori*-associated atrophic gastritis, gastric epithelial cells undergo phosphorylation, which is accompanied by tyrosine protein activity.^[54]^ Tyrosine protein phosphorylation activates extracellular signal-regulated kinase 1/2 (ERK1/2).^[55]^ ERK1/2 affects atherosclerotic progression and reduces atherosclerotic plaque stability.^[56]^ The role of protein tyrosine kinase is broad and its role in these 2 conditions deserves further investigation.

In TF analysis, SPI1 is at the core of the TF regulatory network. SPI1 acts as a key regulator of immune system signaling communication. It is involved in the differentiation of macrophages, B cells, and NK cells, but also regulates gene expression in early T-cell development.^[57,58]^ The expression of SPI1 was significantly increased after a high-fat diet.^[59]^ Inhibition of SPI1-forming complexes leads to a reduction in atherosclerotic lesions, but the exact mechanism is unknown.^[60]^ A study on atherosclerosis and periodontitis revealed that SPI1 plays a pivotal role in both diseases.^[61]^ These findings suggest that SP1, as a crucial TF, exerts a significant influence on chronic inflammatory conditions and warrants further investigation. In our research, SPI1 is associated with several hub genes, such as HCK, ITGB2, and CYBB.HCK is a member of the Src family of tyrosine kinases that plays an important role in immune cell survival, proliferation, migration, and phagocytosis. The Src-like kinase p61HcK is required for the construction of comets, like F-actin structures in lysosomes and at the tip of tyrosine-phosphorylated CagA, and it has been reported to inhibit SKF activation, which triggers proinflammatory and antiapoptotic responses in the gastric epithelium that are chronically detrimental to the human host.^[62]^ Additionally, the role of HCK in the development of atherosclerosis cannot be ignored. A deficiency of hematopoietic function in HCK can reduce the occurrence of atherosclerosis.^[63]^ A bioinformatics analysis showed that HCK expression was elevated in AA plaques, which could distinguish between patients with CAD and healthy individuals.^[64]^ HCK may be a key point of protein tyrosine kinase activity in both diseases, and the exact mechanism of action deserves experimental exploration. ITGB2, also known as leukocyte-specific CD18, is involved in cell adhesion as well as cell surface-mediated signaling and plays an important role in immune responses.^[65]^ In atrophic gastritis caused by *H. pylori*, the expression of adhesion molecules (CD11b/CD18) is increased, and the transendothelial migration leads to capillary blockage and gastric injury.^[66,67]^ ITGB2 also plays a role in cell recruitment during atherosclerosis. ITGB2 encodes leukocyte surface adhesion molecules that directly promote leukocyte transendothelial migration and disrupt endothelial barrier function, a key step in atherogenesis.^[68]^ ITGB2 may play a regulatory role in both diseases by regulating the proliferation and differentiation of immune cells. Functional analysis of CAG and atherosclerosis revealed a high enrichment of reactive oxygen pathways, which may be mediated through CYBB. CYBB (NADPH oxidase 2, NOX2) is expressed in several diseases and is a key component of membrane-bound oxidases in superoxide-producing phagocytes and a good marker of infiltrating inflammatory cells.^[69,70]^ The gastric mucosa initiates an inflammatory response through the activation of NOX2, a step that occurs in atrophic gastritis.^[70,71]^ In the process of atherosclerosis, micro-oxidized low-density lipoprotein stimulates the production of ROS in macrophages by activating NOX2, which promotes the occurrence of atherosclerosis and the progression of atherosclerotic lesions.^[72]^

Previous studies have reported an association between atrophic gastritis and atherosclerosis, bioinformatics approaches have been considered to individually study the pathogenesis of both diseases. However, no bioinformatics approach has been used to explore the common pivotal genes and pathogenesis of the 2 diseases. To the best of our knowledge, the present study has explored a bioinformatics approach for the first time to identify the DEGs, pivotal genes, and TFs shared by the 2 diseases. However, our study has some limitations, and further experiments are needed to validate our results and provide more reliable conclusions.

## 5. Conclusion

Many studies have previously explored the hub genes in CAG and atherosclerosis. Studies have also explored the correlation and possible pathogenesis of the 2 diseases. However, few studies have explored the common molecular mechanisms of atrophic gastritis complicated by atherosclerosis using bioinformatics approaches. We explored and identified for the first time common DEGs, hub genes, and TFs between CAG and atherosclerosis, which will help to elucidate the mechanisms of the 2 diseases and provide potential directions for further study.

## Acknowledgments

We acknowledge GEO database for providing their platforms and contributors for uploading their meaningful datasets. We would like to acknowledge the reviewers and editors for their invaluable comments on this study.

## Author contributions

**Conceptualization:** Xiaoxiao Men.

**Formal analysis:** Xiaoxiao Men, Qianqian Xu.

**Methodology:** Xiaoxiao Men, Mingyue Liu.

**Writing—original draft:** Xiaoxiao Men, Xiuju Shi.

**Data curation:** Xiuju Shi.

**Visualization:** Xiuju Shi, Hongli Yang.

**Writing—review & editing:** Hongwei Xu.

**Validation:** Ling Wang.

**Funding acquisition:** Xiaoju Men.

**Supervision:** Xiaoju Men.

## References

[1] Ling W Li Y Jiang W. Common mechanism of pathogenesis in gastrointestinal diseases implied by consistent efficacy of single Chinese medicine formula: a PRISMA-compliant systematic review and meta-analysis. Medicine (Baltim). 2015;94:e1111. 10.1097/MD.0000000000001111 PMC4504579 26166106

[2] Kotas ME Medzhitov R. Homeostasis, inflammation, and disease susceptibility. Cell. 2015;160:816–27. 25723161 10.1016/j.cell.2015.02.010 PMC4369762

[3] Libby P Ridker PM Maseri A. Inflammation and atherosclerosis. Circulation. 2002;105:1135–43. 11877368 10.1161/hc0902.104353

[4] Strickland RG Mackay IR. A reappraisal of the nature and significance of chronic atrophic gastritis. Am J Dig Dis. 1973;18:426–40. 4573514 10.1007/BF01071995

[5] Barrett TJ. Macrophages in atherosclerosis regression. Arterioscler Thromb Vasc Biol. 2020;40:20–33. 31722535 10.1161/ATVBAHA.119.312802 PMC6946104

[6] Libby P. Inflammation in atherosclerosis. Nature. 2002;420:868–74. 12490960 10.1038/nature01323

[7] Senmaru T Fukui M Tanaka M. Atrophic gastritis is associated with coronary artery disease. J Clin Biochem Nutr. 2012;51:39–41. 22798711 10.3164/jcbn.11-106 PMC3391861

[8] Kutluana U Simsek I Akarsu M. Is there a possible relation between atrophic gastritis and premature atherosclerosis? Helicobacter. 2005;10:623–9. 16302990 10.1111/j.1523-5378.2005.00356.x

[9] Torisu T Takata Y Ansai T. Possible association of atrophic gastritis and arterial stiffness in healthy middle-aged Japanese. J Atheroscler Thromb. 2009;16:691–7. 19729867 10.5551/jat.943

[10] Witherell HL Smith KL Friedman GD. C-reactive protein, *Helicobacter pylori, Chlamydia pneumoniae*, cytomegalovirus and risk for myocardial infarction. Ann Epidemiol. 2003;13:170–7. 12604160 10.1016/s1047-2797(02)00276-4

[11] Zhu F Zuo L Hu R. A ten-genes-based diagnostic signature for atherosclerosis. BMC Cardiovasc Disord. 2021;21:513. 34688276 10.1186/s12872-021-02323-9 PMC8540101

[12] Krawczynska A Herman AP Antushevich H. The influence of photoperiod on the action of exogenous leptin on gene expression of proinflammatory cytokines and their receptors in the thoracic Perivascular Adipose Tissue (PVAT) in ewes. Mediators Inflamm. 2019;2019:7129476. 31780867 10.1155/2019/7129476 PMC6875191

[13] Drici Ael M Moulessehoul S Tifrit A. Effect of IL-1beta and IL-1RN polymorphisms in carcinogenesis of the gastric mucosa in patients infected with *Helicobacter pylori* in Algeria. Libyan J Med. 2016;11:31576. 27340011 10.3402/ljm.v11.31576 PMC4919366

[14] Bashour L Khattab R Harfoush E. The role of interleukin-1 genotype in the association between coronary heart disease and periodontitis in a Syrian population. ISRN Dent. 2013;2013:195678. 23691333 10.1155/2013/195678 PMC3649497

[15] Yang WS SriRamaratnam R Welsch ME. Regulation of ferroptotic cancer cell death by GPX4. Cell. 2014;156:317–31. 24439385 10.1016/j.cell.2013.12.010 PMC4076414

[16] Thorn CF Grosser T Klein TE. PharmGKB summary: very important pharmacogene information for PTGS2. Pharmacogenet Genomics. 2011;21:607–13. 21063235 10.1097/FPC.0b013e3283415515 PMC3141084

[17] Liu Y Neogi A Mani A. The role of Wnt signalling in development of coronary artery disease and its risk factors. Open Biol. 2020;10:200128. 33081636 10.1098/rsob.200128 PMC7653355

[18] Goracy I Kaczmarczyk M Ciechanowicz A. Polymorphism of interleukin 1B may modulate the risk of ischemic stroke in polish patients. Medicina (Kaunas). 2019;55:558. 31480765 10.3390/medicina55090558 PMC6780056

[19] Toyoshima O Nishizawa T Sekiba K. A single nucleotide polymorphism in prostate stem cell antigen is associated with endoscopic grading in Kyoto classification of gastritis. J Clin Biochem Nutr. 2021;68:73–7. 33536715 10.3164/jcbn.20-67 PMC7844668

[20] Ganguly K Rauth S Marimuthu S. Unraveling mucin domains in cancer and metastasis: when protectors become predators. Cancer Metastasis Rev. 2020;39:647–59. 32488403 10.1007/s10555-020-09896-5 PMC7487023

[21] Tin A Kottgen A Folsom AR. Genetic loci for serum magnesium among African-Americans and gene-environment interaction at MUC1 and TRPM6 in European-Americans: the Atherosclerosis Risk in Communities (ARIC) study. BMC Genet. 2015;16:56. 26058915 10.1186/s12863-015-0219-7 PMC4462077

[22] Park JM Han YM Hwang SJ. Therapeutic effects of placenta derived-, umbilical cord derived-, and adipose tissue derived-mesenchymal stem cells in chronic *Helicobacter pylori* infection: comparison and novel mechanisms. J Clin Biochem Nutr. 2021;69:188–202. 34616110 10.3164/jcbn.20-151 PMC8482378

[23] Metghalchi S Ponnuswamy P Simon T. Indoleamine 2,3-dioxygenase fine-tunes immune homeostasis in atherosclerosis and colitis through repression of interleukin-10 production. Cell Metab. 2015;22:460–71. 26235422 10.1016/j.cmet.2015.07.004

[24] Kumar S Kumari N Mittal RD. Association between pro-(IL-8) and anti-inflammatory (IL-10) cytokine variants and their serum levels and *H. pylori*-related gastric carcinogenesis in northern India. Meta Gene. 2015;6:9–16. 26380815 10.1016/j.mgene.2015.07.008 PMC4556814

[25] Arbiser JL Nowak R Michaels K. Evidence for biochemical barrier restoration: topical solenopsin analogs improve inflammation and acanthosis in the KC-Tie2 mouse model of psoriasis. Sci Rep. 2017;7:11198. 28894119 10.1038/s41598-017-10580-y PMC5593857

[26] Schiopu A Cotoi OS. S100A8 and S100A9: DAMPs at the crossroads between innate immunity, traditional risk factors, and cardiovascular disease. Mediators Inflamm. 2013;2013:828354. 24453429 10.1155/2013/828354 PMC3881579

[27] Grubisa I Otasevic P Vucinic N. Combined GSTM1 and GSTT1 null genotypes are strong risk factors for atherogenesis in a Serbian population. Genet Mol Biol. 2018;41:35–40. 29658969 10.1590/1678-4685-GMB-2017-0034 PMC5901493

[28] You WC Hong JY Zhang L. Genetic polymorphisms of CYP2E1, GSTT1, GSTP1, GSTM1, ALDH2, and ODC and the risk of advanced precancerous gastric lesions in a Chinese population. Cancer Epidemiol Biomarkers Prev. 2005;14:451–8. 15734972 10.1158/1055-9965.EPI-04-0311

[29] Kim J Cha YN Surh YJ. A protective role of nuclear factor-erythroid 2-related factor-2 (Nrf2) in inflammatory disorders. Mutat Res. 2010;690:12–23. 19799917 10.1016/j.mrfmmm.2009.09.007

[30] Molani Gol R Rafraf M Asghari Jafarabadi M. Evaluation of cardiovascular risk factors in women referring to health centers in Tabriz, Iran, 2017. Health Promot Perspect. 2018;8:315–22. 30479987 10.15171/hpp.2018.45 PMC6249489

[31] Swiatkowski M Budzynski J Klopocka M. Oxygen metabolism disturbances in the pathogenesis of gastric and duodenal diseases. Przegl Lek. 1999;56:220–6. 10442013

[32] Huang da W Sherman BT Lempicki RA. Systematic and integrative analysis of large gene lists using DAVID bioinformatics resources. Nat Protoc. 2009;4:44–57. 19131956 10.1038/nprot.2008.211

[33] Szklarczyk D Gable AL Lyon D. STRING v11: protein–protein association networks with increased coverage, supporting functional discovery in genome-wide experimental datasets. Nucleic Acids Res. 2019;47:D607–13. 30476243 10.1093/nar/gky1131 PMC6323986

[34] Warde-Farley D Donaldson SL Comes O. The GeneMANIA prediction server: biological network integration for gene prioritization and predicting gene function. Nucleic Acids Res. 2010;38:W214–220. 20576703 10.1093/nar/gkq537 PMC2896186

[35] Wang C Wei Y Zhang L. Blood flow differences in cun-kou (radial) artery and anterior tibial artery: normal people vs patients with chronic gastritis. J Tradit Chin Med. 2018;38:911–6. 32186139

[36] Sadjadi A Yazdanbod A Lee YY. Serum ghrelin; a new surrogate marker of gastric mucosal alterations in upper gastrointestinal carcinogenesis. PLoS One. 2013;8:e74440. 24098650 10.1371/journal.pone.0074440 PMC3787044

[37] Santarelli L Gabrielli M Cremonini F. Atrophic gastritis as a cause of hyperhomocysteinaemia. Aliment Pharmacol Ther. 2004;19:107–11. 14687172 10.1046/j.1365-2036.2003.01820.x

[38] Saijo Y Utsugi M Yoshioka E. Inflammation as a cardiovascular risk factor and pulse wave velocity as a marker of early-stage atherosclerosis in the Japanese population. Environ Health Prev Med. 2009;14:159–64. 19568843 10.1007/s12199-009-0080-2 PMC2684804

[39] Ai W Wu M Chen L. Ghrelin ameliorates atherosclerosis by inhibiting endoplasmic reticulum stress. Fundam Clin Pharmacol. 2017;31:147–54. 27753125 10.1111/fcp.12251

[40] Li WG Gavrila D Liu X. Ghrelin inhibits proinflammatory responses and nuclear factor-kappaB activation in human endothelial cells. Circulation. 2004;109:2221–6. 15117840 10.1161/01.CIR.0000127956.43874.F2

[41] Eun Bae S Hoon Lee J Soo Park Y. Decrease of serum total ghrelin in extensive atrophic gastritis: comparison with pepsinogens in histological reference. Scand J Gastroenterol. 2016;51:137–44. 26513345 10.3109/00365521.2015.1083049

[42] Rodriguez-Castro KI Franceschi M Noto A. Clinical manifestations of chronic atrophic gastritis. Acta Biomed. 2018;89(8-S):88–92. 30561424 10.23750/abm.v89i8-S.7921 PMC6502219

[43] Ni T Gao F Zhang J. Impaired autophagy mediates hyperhomocysteinemia-induced HA-VSMC phenotypic switching. J Mol Histol. 2019;50:305–14. 31028566 10.1007/s10735-019-09827-x

[44] Spence JD. Homocysteine lowering for stroke prevention: unravelling the complexity of the evidence. Int J Stroke. 2016;11:744–7. 27462097 10.1177/1747493016662038

[45] Wu X Zhang L Miao Y. Homocysteine causes vascular endothelial dysfunction by disrupting endoplasmic reticulum redox homeostasis. Redox Biol. 2019;20:46–59. 30292945 10.1016/j.redox.2018.09.021 PMC6174864

[46] Zhang Z Wei C Zhou Y. Homocysteine induces apoptosis of human umbilical vein endothelial cells via mitochondrial dysfunction and endoplasmic reticulum stress. Oxid Med Cell Longev. 2017;2017:5736506. 28630659 10.1155/2017/5736506 PMC5467318

[47] Mo L Ma C Wang Z. Integrated bioinformatic analysis of the shared molecular mechanisms between osteoporosis and atherosclerosis. Front Endocrinol (Lausanne). 2022;13:950030. 35937806 10.3389/fendo.2022.950030 PMC9353191

[48] Schumacher MA Donnelly JM Engevik AC. Gastric Sonic Hedgehog acts as a macrophage chemoattractant during the immune response to *Helicobacter pylori*. Gastroenterology. 2012;142:1150–9.e6. 22285806 10.1053/j.gastro.2012.01.029 PMC3335966

[49] Waghray M Zavros Y Saqui-Salces M. Interleukin-1beta promotes gastric atrophy through suppression of Sonic Hedgehog. Gastroenterology. 2010;138:562–72 [572.e1]. 19883649 10.1053/j.gastro.2009.10.043 PMC2895809

[50] Gerhardt T Ley K. Monocyte trafficking across the vessel wall. Cardiovasc Res. 2015;107:321–30. 25990461 10.1093/cvr/cvv147 PMC4592323

[51] Candon S McHugh RS Foucras G. Spontaneous organ-specific Th2-mediated autoimmunity in TCR transgenic mice. J Immunol. 2004;172:2917–24. 14978094 10.4049/jimmunol.172.5.2917

[52] Harris PR Wright SW Serrano C. *Helicobacter pylori* gastritis in children is associated with a regulatory T-cell response. Gastroenterology. 2008;134:491–9. 18242215 10.1053/j.gastro.2007.11.006

[53] Tedgui A Mallat Z. Cytokines in atherosclerosis: pathogenic and regulatory pathways. Physiol Rev. 2006;86:515–81. 16601268 10.1152/physrev.00024.2005

[54] Suzuki M Mimuro H Kiga K. *Helicobacter pylori* CagA phosphorylation-independent function in epithelial proliferation and inflammation. Cell Host Microbe. 2009;5:23–34. 19154985 10.1016/j.chom.2008.11.010

[55] Greig FH Kennedy S Gibson G. PEA-15 (Phosphoprotein Enriched in Astrocytes 15) is a protective mediator in the vasculature and is regulated during neointimal hyperplasia. J Am Heart Assoc. 2017;6:e006936. 28893763 10.1161/JAHA.117.006936 PMC5634313

[56] Blanks AM Pedersen LN Bohmke N. Sex differences in monocyte CCR2 expression and macrophage polarization following acute exercise. Life Sci. 2022;299:120557. 35447130 10.1016/j.lfs.2022.120557

[57] Guerriero A Langmuir PB Spain LM. PU.1 is required for myeloid-derived but not lymphoid-derived dendritic cells. Blood. 2000;95:879–85. 10648399

[58] Oikawa T Yamada T Kihara-Negishi F. The role of Ets family transcription factor PU.1 in hematopoietic cell differentiation, proliferation and apoptosis. Cell Death Differ. 1999;6:599–608. 10453070 10.1038/sj.cdd.4400534

[59] Pan Y Yu C Huang J. Bioinformatics analysis of vascular RNA-seq data revealed hub genes and pathways in a novel Tibetan minipig atherosclerosis model induced by a high fat/cholesterol diet. Lipids Health Dis. 2020;19:54. 32213192 10.1186/s12944-020-01222-w PMC7098151

[60] Zhao L Cuff CA Moss E. Selective interleukin-12 synthesis defect in 12/15-lipoxygenase-deficient macrophages associated with reduced atherosclerosis in a mouse model of familial hypercholesterolemia. J Biol Chem. 2002;277:35350–6. 12122008 10.1074/jbc.M205738200

[61] Zeng Y Cao S Chen M. Integrated analysis and exploration of potential shared gene signatures between carotid atherosclerosis and periodontitis. BMC Med Genomics. 2022;15:227. 36316672 10.1186/s12920-022-01373-y PMC9620656

[62] Oldani A Cormont M Hofman V. *Helicobacter pylori* counteracts the apoptotic action of its VacA toxin by injecting the CagA protein into gastric epithelial cells. PLoS Pathog. 2009;5:e1000603. 19798427 10.1371/journal.ppat.1000603 PMC2745580

[63] Medina I Cougoule C Drechsler M. Hck/Fgr kinase deficiency reduces plaque growth and stability by blunting monocyte recruitment and intraplaque motility. Circulation. 2015;132:490–501. 26068045 10.1161/CIRCULATIONAHA.114.012316 PMC4535360

[64] Liu C Zhang H Chen Y. Identifying RBM47, HCK, CD53, TYROBP, and HAVCR2 as hub genes in advanced atherosclerotic plaques by network-based analysis and validation. Front Genet. 2020;11:602908. 33519905 10.3389/fgene.2020.602908 PMC7844323

[65] Ostermann G Weber KS Zernecke A. JAM-1 is a ligand of the beta(2) integrin LFA-1 involved in transendothelial migration of leukocytes. Nat Immunol. 2002;3:151–8. 11812992 10.1038/ni755

[66] Hatz RA Meimarakis G Lehn N. Granulocyte activation by *Helicobacter pylori*. Eur J Med Res. 1996;1:537–42. 9438157

[67] Yoshida N Yoshikawa T. Effect of *Helicobacter pylori*-mediated inflammation on nonsteroidal anti-inflammatory drugs-induced gastric mucosal injury. Keio J Med. 2002;51:45–50. 12528937 10.2302/kjm.51.supplement2_45

[68] Rysa J. Novel insights into the molecular basis of calcific aortic valve disease. J Thorac Dis. 2020;12:6419–21. 33282343 10.21037/jtd-20-1669 PMC7711394

[69] Kim D You B Jo EK. NADPH oxidase 2-derived reactive oxygen species in spinal cord microglia contribute to peripheral nerve injury-induced neuropathic pain. Proc Natl Acad Sci U S A. 2010;107:14851–6. 20679217 10.1073/pnas.1009926107 PMC2930447

[70] Li H Zhou Y Zheng Y. The gastric mucosa from patients infected with CagA+ or VacA+ *Helicobacter pylori* has a lower level of dual oxidase-2 expression than uninfected or infected with CagA−/VacA− *H. pylori*. Dig Dis Sci. 2016;61:2328–37. 27048452 10.1007/s10620-016-4144-z PMC4943970

[71] Bulua AC Simon A Maddipati R. Mitochondrial reactive oxygen species promote production of proinflammatory cytokines and are elevated in TNFR1-associated periodic syndrome (TRAPS). J Exp Med. 2011;208:519–33. 21282379 10.1084/jem.20102049 PMC3058571

[72] Bae YS Lee JH Choi SH. Macrophages generate reactive oxygen species in response to minimally oxidized low-density lipoprotein: toll-like receptor 4- and spleen tyrosine kinase-dependent activation of NADPH oxidase 2. Circ Res. 2009;104:210–8 [21 p following 218]. 19096031 10.1161/CIRCRESAHA.108.181040 PMC2720065

